# Determination of the Total Mass of Antioxidant Substances and Antioxidant Capacity per Unit Mass in Serum Using Redox Titration

**DOI:** 10.1155/2014/928595

**Published:** 2014-07-20

**Authors:** Meijuan Zhang, Na Liu, Hui Liu

**Affiliations:** College of Medical Laboratory, Dalian Medical University, Dalian 116044, China

## Abstract

*Objective*. Total antioxidant capacity in serum is determined by the total mass of antioxidant substances and the antioxidant capacity per unit mass (average activity). The purpose of this study was to develop a method to determine the mass of antioxidant substances and average activity in human serum. *Methods*. Specimens of serum were collected from 100 subjects each from two different age groups: over 75 years old and 20–40 years old. The test serum was diluted into a series of concentrations, following which standard oxidation agents (KMnO_4_ for potassium permanganate method and I_2_ for iodimetry) were added to each concentration of serum, and the absorbance of the mixture (optical density, OD) was measured. The OD value and logarithm of dilution factor (lgT) at the end of the titration were obtained, from which the lgT could be considered as mass of antioxidant substances (M). Total antioxidant capacity (Ta) was calculated with the equation Ta = 100/(OD1 + 2 ∗ OD2 + 2 ∗ OD3 + 2 ∗ OD4 + OD5), and average activity (A) was calculated as A = Ta/M. *Results*. The potassium permanganate method generated similar results to the iodimetric method. Compared with the younger group, total antioxidant capacity in the over-75-year age group was found to be significantly reduced, along with a decrease in the mass of antioxidant substances and average activity levels in human serum. *Conclusions*. The approach described in this paper is suitable for determining the average activity and mass of antioxidant substances in human serum.

## 1. Introduction

The antioxidant capacity of the human body is closely linked to health, where the total antioxidant capacity of human serum is made up of enzymatic and nonenzymatic components. Oxidative stress occurs when the balance is disrupted between the production of reactive oxygen species (ROS) and endogenous antioxidant mechanisms to counteract the effects of ROS or repair resulting in damage [1]. Most studies have focused on the antioxidant capacity of tissues by determining the activities of the body's main antioxidases or levels of low-molecular-weight nonenzymatic antioxidants [2–4]. When antioxidant functions are reduced, the degree of free radical damage is more severe. Oxidative stress is a key feature of severe cardiovascular disease, as ROS are involved in all disease stages, from endothelial dysfunction to atheromatous plaque formation [5]. Accumulating evidence has indicated that abnormal antioxidant capacity in humans is closely related to cardiovascular and renal disorders, gout, obesity, and diabetes mellitus type 2 [5–10]. Therefore, determining the antioxidant capacity of the body has become of major interest in medical research.

As the antioxidant material in serum is a mix of components, total antioxidant capacity is generally used as an indicator of the antioxidant capacity of the body, reflecting the sum of all the antioxidant substances present in the serum [11–13]. The antioxidant capacity per unit mass can be obtained by dividing the total antioxidant capacity by the total mass of all substances. Here, we define the antioxidant capacity per unit mass as the average activity of the antioxidant substances. Therefore, a reduction in the total antioxidant capacity of serum can be caused by a decrease in the total mass of antioxidant substances in the serum, a reduction in their average activity, or a combination of both. Thus, it is necessary to determine the total antioxidant capacity of human serum, the mass of antioxidant substances present, and the average activity of these substances.

Several accepted methods of evaluating antioxidant capacity exist* in vitro*, such as iron ion reduction/oxidation resistance measurements, oxygen-free radical absorption ability measurements, and chemiluminescence [14]. However, these most popular methods for measuring serum antioxidant capacity cannot determine the mass of antioxidants present or the average activity of these substances. This paper proposes a method to determine the activity and mass of antioxidant substances in serum using redox titration with a constant standard oxidant. The potassium permanganate and iodimetric methods are suitable methods for redox titration. The mass of antioxidants will gradually be reduced in a series of diluted test specimens; the antioxidants originally present will almost disappear following the first dilution, and this dilution can be considered as an indicator of antioxidant substances. The average activity of the antioxidant substances can be obtained from dividing the total antioxidant capacity by the mass of antioxidant substances. We also included subjects from two different age groups in our study to test this method for determining the total antioxidant capacity, mass of antioxidants, and average activity. This technique has potential applications in clinical research regarding antioxidant defense and oxidative stress.

## 2. Materials and Methods

### 2.1. Serum Specimens

Serum specimens were randomly collected from 50 healthy people over the age of 75 years (25 males and 25 females; mean age 82.0 ± 4.4 years) and from a separate group of 50 healthy subjects aged 20 to 40 years (25 males and 25 females; mean age 35.5 ± 2.7 years). These groups were treated, respectively, as the over-75 (older) group and the control (younger) group. All subjects were northern Han Chinese. This study protocol was approved by the Ethical Committee of Dalian Medical University.

### 2.2. Potassium Permanganate Method

The test serum was diluted with distilled water into a series of concentrations (1 : 10; 1 : 20; 1 : 40; 1 : 80; and 1 : 160). For each concentration of serum, a 20 *μ*L aliquot was taken and added to a 96-well ELISA plastic plate. A blank control (no serum) was established.

To prepare a 5 mmol/L solution of KMnO_4_, we took 79 mg of KMnO_4_ and dissolved it in 100 mL distilled water. Aliquots of 100 *μ*L of this solution were then added to the plastic plate containing the serum specimens and mixed uniformly by shaking.

The mixture was warmed for 30 min in a water bath at 37°C, after which an enzyme-labeling instrument was used to determine the absorbance of the mixture (optical density, OD, of 570 nm).

### 2.3. Iodimetry

To prepare 50 mmol/L iodine stock solution and 0.25 mmol/L working solution, we took iodine tablets (6.5 g, sublimated) and potassium iodide (17.5 g) dissolved in deionized water (500 mL) as stock solutions; working solutions were prepared by diluting the stock solution (1 mL) with deionized water (199 mL). A 5 g/L starch solution was prepared by taking 0.5 g of soluble starch and dissolving it in 100 mL distilled water. An equivalent volume of starch solution was added to the iodine solution as a chromogenic indicator to obtain a blue-black iodine solution.

The method used to dilute the test serum was similar to that of the potassium permanganate method. For each concentration of serum, a 20 *μ*L aliquot was taken and added to a 96-well ELISA plastic plate. A 200 *μ*L aliquot of the blue-black iodine solution was then added to each serum sample in the plastic plate and mixed uniformly by shaking. The mixture was warmed for 30 min in a water bath at 37°C, following which an enzyme-labeling instrument was used to determine the absorbance of the solution (OD of 570 nm).

### 2.4. Calculation of Total Antioxidant Capacity of Serum

**Figure 1:**
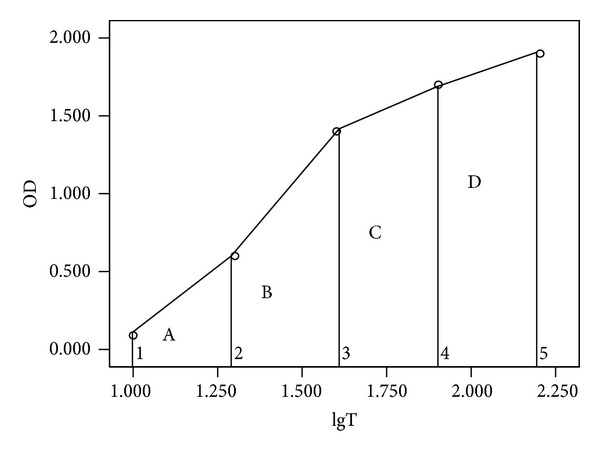
Schematic diagram of total antioxidant capacity, calculated as the total area under the plotted line.

A constant volume of standard oxidation agent (iodine or potassium permanganate) was added to a series of diluted test samples (double dilution), and measurements were performed in a point-by-point manner. Changes in OD values were arranged from the smallest to the largest. OD values changed as a result of oxidation agents reacting with reducing serum samples. The serum dilution factors (1 : 10; 1 : 20; 1 : 40; 1 : 80; and 1 : 160) were transformed into a logarithmic scale (1.0, 1.30, 1.6, 1.9, and 2.2) to simple calculations (Figure 1). The area under the plotted line inversely represented the total antioxidant capacity of the serum, as explained below.

The total area was obtained by summing the series of smaller areas formed under the curve (*A* + *B* + *C* + *D*). For example, the area A representing dilution factors of 10 and 20 could be regarded as the area of the trapezoid bounded by OD1, OD2, lgT1, and lgT2, where the square (*S*) could be calculated as follows:

(1) $$\begin{array}{c} S_{A}=\left(\text{OD}1+\text{OD}2\right)*\frac{\left(\text{lgT}2-\text{lgT}1\right)}{2}. \end{array}$$

For this series of dilutions, the dilution interval was constant, meaning that (lgT2 − lgT1)/2 was a constant and so it could be ignored. Therefore, *S*1 = (OD1 + OD2) and the total area was equal to

(2) S=SA+SB+SC+SD=(OD1+OD2)+(OD2+OD3) +(OD3+OD4)+(OD4+OD5)=OD1+2×(OD2+OD3+OD4)+OD5.

Here, the larger the area, the lower the total antioxidant capacity of the serum. In other words, total antioxidant capacity was expressed as the reciprocal of the area under the curve. To better present this figure, the numerator was multiplied by 100 as follows:

(3) $$\begin{array}{c} \text{Ta}=\frac{100}{\left[\text{OD}1+2\times \left(\text{OD}2+\text{OD}3+\text{OD}4\right)+\text{OD}5\right]}, \end{array}$$

where Ta represented the total antioxidant capacity; the larger the Ta, the larger the total antioxidant capacity of the serum.

### 2.5. Calculation of Mass of Antioxidants in the Serum

**Figure 2:**
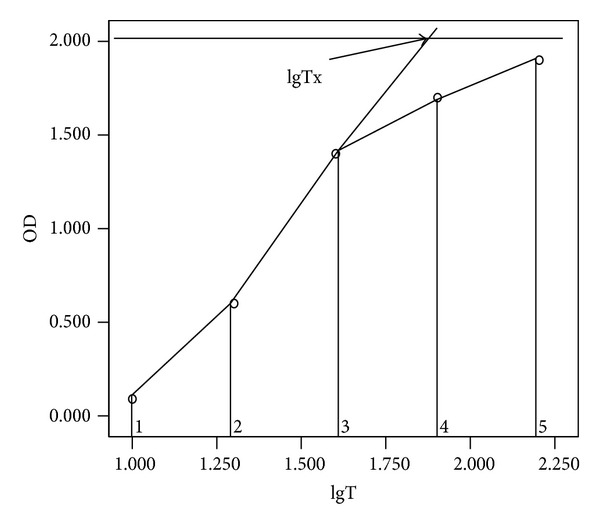
Schematic diagram for the calculation of serum antioxidant mass. lgTx represents the crossing point between lines of jump range and the blank control; lgTx value on the *x*-axis represents the mass of antioxidants in the serum.

A constant volume of a standard oxidation agent was added to a series of diluted test specimens. The absorbance values (OD values) of the specimens were arranged from the smallest to the largest. OD values of adjacent concentrations were subtracted, and the largest absolute value of the difference was defined as the titration jump range. A linear regression analysis was obtained with lower and higher values beside the jump range and lgTx, respectively, and a crossed point between lines of jump range and blank control (Figure 2) was calculated as follows:

(4) $$\begin{array}{c} M=0.3\times \frac{\left(\text{ODcontrol}-\text{ODlow}\right)}{\left(\text{ODhigh}-\text{ODlow}\right)}+\text{lgTlow}, \end{array}$$

where the *M* represented the titration of antioxidant activity that had disappeared and the largest logarithmic dilution factor (*M* value) implied the larger mass of antioxidants in the serum; ODcontrol represented the OD value of the blank control; ODhigh and ODlow represented higher and lower OD values beside the jump range; lgTlow represented the lowest logarithm of the dilution beside the jump range.

For serum samples diluted to concentrations of 1 : 10, 1 : 20, 1 : 40, 1 : 80, and 1 : 160, OD values were 0.095, 0.496, 1.176, 1.682, and 1.986, respectively; the OD value of the blank control was 2.07. The jump range was 1 : 20 to 1 : 40, where the mass of antioxidant substances was calculated to be

(5) 0.3×(2.07−0.496)(1.176−0.496)+lg20=1.99.

### 2.6. Calculation of Average Activity of Antioxidants in the Serum

The average activity (*A*) of the antioxidant substances was obtained by dividing the total antioxidant capacity (Ta) by the mass of antioxidant substances (*M*):

(6) $$\begin{array}{c} A=\frac{\text{Ta}}{M}. \end{array}$$

### 2.7. Statistical Analysis

The independent-sample* t*-test was used to assess the difference between the means of two groups. The strength of the relationships between results from two methods was assessed by the Pearson correlation test. The above analyses were implemented using SPSS statistical analysis software (SPSS Inc., Chicago, IL). *P*  values less than 0.05 (two-tailed) were considered significant.

## 3. Results

For the potassium permanganate method, the titration jump range of the 100 test specimens was between 1 : 10 and 1 : 20, while the jump range for the iodimetric method was between 1 : 20 and 1 : 40.

Total antioxidant capacity, average activity, and mass of antioxidant substances in the over-75 group and the control group are shown in Tables 1 and 2, respectively. The potassium permanganate method generated similar results to those produced by the iodimetric method. Compared with the control group, the total antioxidant capacity in the serum of participants from the over-75-year group was found to be significantly reduced (*P* < 0.05), while the average activity of the antioxidant substances significantly decreased (*P* < 0.05), and the mass of antioxidant substances significantly also decreased (*P* < 0.05).

**Figure 3:**
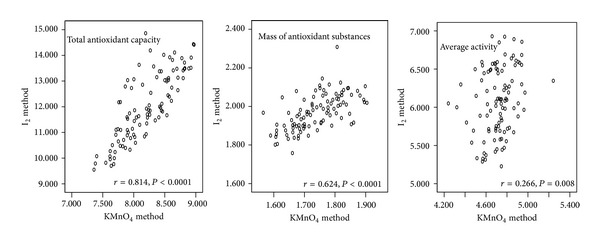
Correlation of potassium permanganate method with iodimetric method for determination of total antioxidant capacity, average serum antioxidant activity, and mass of antioxidants in serum.

For the determination of the total antioxidant capacity, the average activity of antioxidant substances, and the mass of antioxidant substances, the potassium permanganate method was found to be significantly correlated with iodimetry (Figure 3).

## 4. Discussion

The potassium permanganate and iodimetric methods are classic redox titration techniques with well-known and reliable titration principles. For the determination of serum specimens here, we used neutral titration conditions to adequately maintain the activity of organic antioxidants to ensure that the test conditions roughly matched intracorporeal conditions, although the reactions are complex in neutral conditions for these two methods. The different methods differ in terms of assay principles and experimental conditions, and the degree of interference from serum components could be different. It was therefore necessary to use two methods to achieve a cross-validation. Comparative analyses using the potassium permanganate and iodimetric methods were strongly correlated, implying that these were effective measurement methods without significant levels of interference from the serum components. As the current methods of evaluating antioxidant capacity require acidic conditions, we established an assay to measure the total mass of antioxidant substances and antioxidant capacity per unit mass in serum based on classic potassium permanganate and iodimetric methods instead. Methods for the determination of special substances, such as oxygen radical absorbance capability, hydroxyl radical scavenger activity, superoxide dismutase, catalase, and glutathione peroxidase, remained unsuitable for the establishment of an assay to measure the total mass of antioxidant substances and antioxidant capacity per unit mass.

A sample set comprising a series of concentrations was prepared. OD value changes were arranged from the smallest to the largest. Compared to the traditional methods, five OD values were considered simultaneously in our assay. Therefore, we used the area below the reaction curve to inversely represent the total antioxidant capacity of serum. We found that the total antioxidant capacity of serum in the over-75 group was significantly lower than that of the control group.

Continuous dilution gradually reduced antioxidant levels in the serum. Antioxidants originally present at low levels disappeared following the first dilution and antioxidant activity was found to be zero. Therefore, the titration jump range can be understood as changes in the activity of the main antioxidant substances in serum from low levels to a concentration below the limit of detection. However, some antioxidants originally present at high levels remained at relatively high levels at higher dilution factors. Therefore, a value of crossed point between lines of jump range and blank control on *x*-axis represnts the mass of antioxidants present. It is feasible to obtain the average activity of antioxidant substances in the serum by dividing total antioxidant capacity by the mass of antioxidants present.

In our experiment the results generated by the potassium permanganate method significantly correlated with those produced by the iodimetric method. The two methods effectively verified each other, suggesting that the calculation model is reasonable and the titration techniques are reliable.

The human body hosts a complex antioxidant system to prevent oxidative damage [15–17]. Compared with the control group, the total antioxidant capacity of serum in the >75-year age group was significantly reduced along with a decrease of the mass of antioxidant substances and average activity in human serum. This finding suggested that the decrease in total antioxidant capacity was linked to the drop in the total mass of antioxidant substances and average activity in the >75-year age group. The identity of antioxidant substances that decrease and the details of the underlying mechanisms both require further study.

Abnormal antioxidant capacity is linked to cardiovascular and cerebrovascular diseases, tumors, atherosclerosis, diabetes mellitus, and central nervous system diseases amongst others [18–20]. Concentrating solely on total antioxidant capacity may overlook some essential constituent elements of antioxidant capacity. Our method offers potential new measures and experimental indicators for research into a range of diseases and has potential clinical application.

## Figures and Tables

**Table 1:** Total antioxidant capacity (Ta), average activity (A), and mass of antioxidant substances (M) in serum of two different groups (potassium permanganate method).

Group	Antioxidant capacity in serum		
	Ta	A	M
Old	8.067 ± 0.405	4.684 ± 0.175	1.723 ± 0.085
Young	8.341 ± 0.373	4.750 ± 0.112	1.756 ± 0.072

*t* value	3.525	2.254	2.092
*P* value	0.001	0.026	0.039

**Table 2:** Total antioxidant capacity (Ta), average activity (A), and mass of antioxidant substances (M) in serum of two different groups (iodimetric method).

Group	Antioxidant capacity in serum		
	Ta	A	M
Old	11.689 ± 1.266	5.990 ± 0.421	1.947 ± 0.083
Young	12.363 ± 1.311	6.183 ± 0.437	1.995 ± 0.096

*t* value	2.617	2.251	2.726
*P* value	0.010	0.027	0.008
